# Ultrarapid Development of Ruptured Esophageal Varices in a Patient With a History of Heller Myotomy

**DOI:** 10.7759/cureus.58954

**Published:** 2024-04-24

**Authors:** Binyamin R Abramowitz, Rachel R Meier, Michelle Chen, Suzette Graham-Hill

**Affiliations:** 1 Internal Medicine, State University of New York Downstate Health Sciences University, Brooklyn, USA; 2 Dentistry, Columbia University, New York, USA; 3 Cardiology, State University of New York Downstate Health Sciences University, Brooklyn, USA

**Keywords:** endoscopy, hematemesis, cirrhosis, varices, esophagus

## Abstract

Esophageal varices commonly affect cirrhotic patients as a result of elevated portal system resistance. Blood pools within esophageal portosystemic collateral vessels, which can eventually rupture, leading to life-threatening hemorrhage. To prevent this, cirrhotic patients without a history of varices undergo endoscopic surveillance for varices every 2-3 years. We present an unusual case of variceal hemorrhage in a patient who was seen to have no varices on endoscopic evaluation only a month earlier.

## Introduction

Esophageal varices are enlarged veins within the esophagus, connecting the portal and systemic circulation. In cirrhotic patients, resistance within the portal circulation can often lead to pooling of blood within these esophageal portosystemic collateral vessels, which can eventually lead to rupture, and a deadly upper gastrointestinal bleed [1]. To screen for esophageal varices, patients with cirrhosis typically undergo surveillance endoscopy every 1-3 years [2]. We present an unusual case of variceal hemorrhage in a patient who was seen to have no varices on esophagogastroduodenoscopy (EGD) only a month earlier.

This article was previously presented as a meeting abstract at the SAGES (Society of American Gastrointestinal and Endoscopic Surgeons) 2023 annual meeting on March 29, 2023. 

## Case presentation

**Figure 1:**
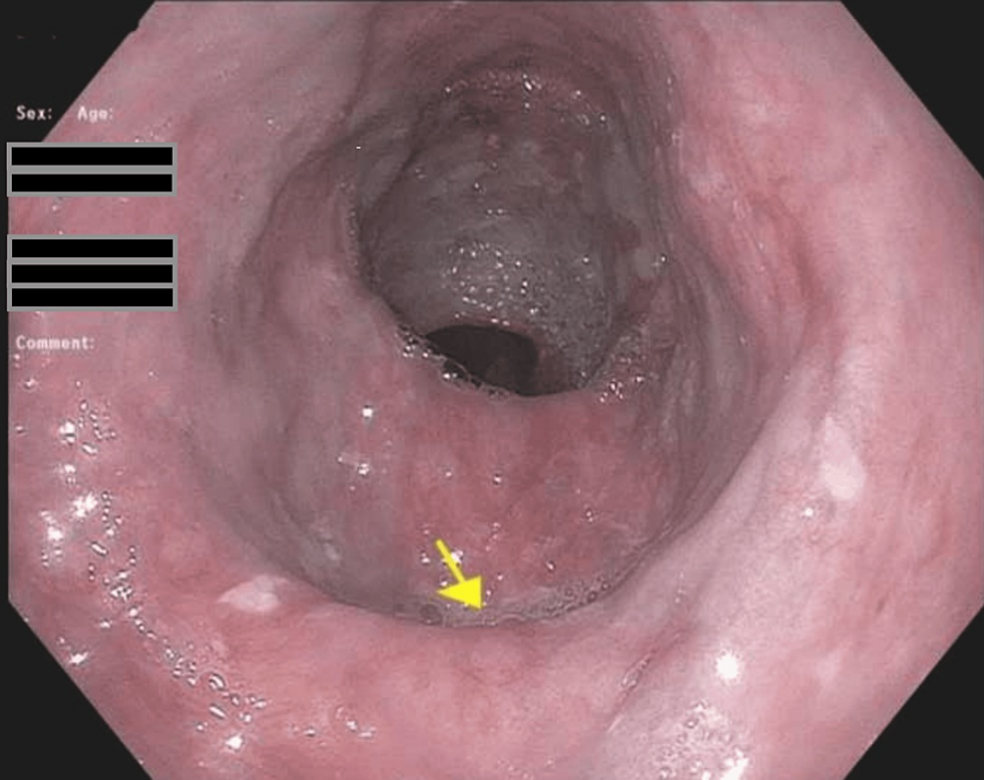
Patient’s initial esophagogastroduodenoscopy showing lower esophagus with no varices present. The yellow arrow points to the diverticulum by the gastroesophageal junction.

**Figure 2:**
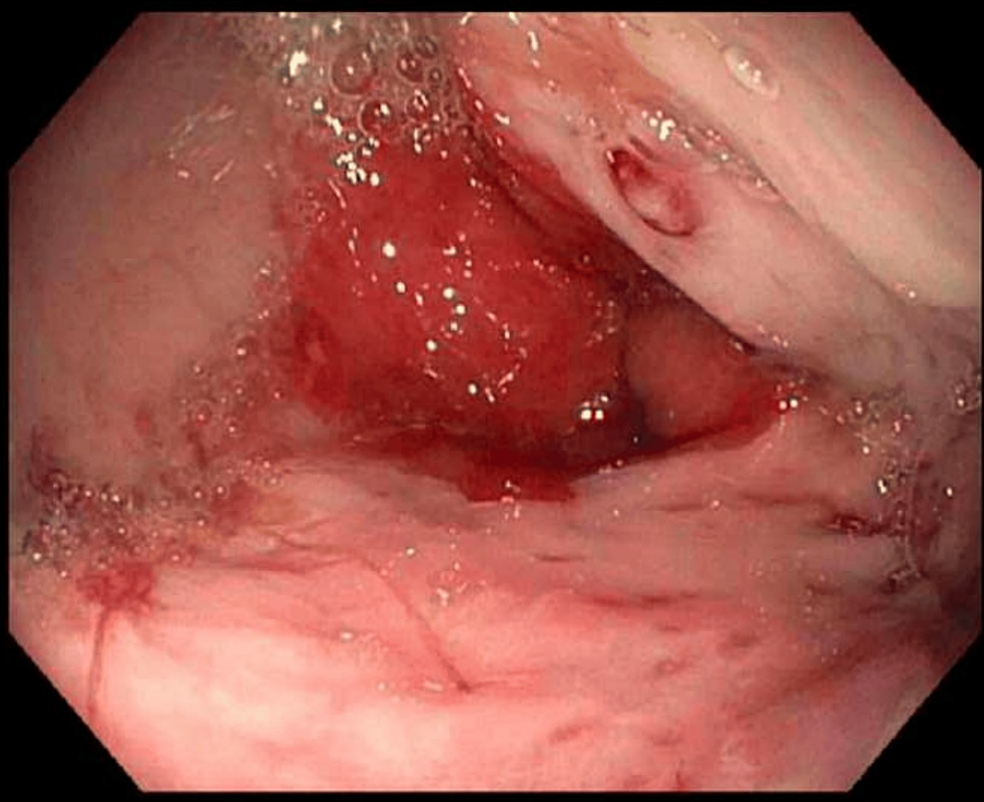
Esophagogastroduodenoscopy during patient’s hospital admission for hematemesis, showing white nipple sign with small columns of varices.

A 62-year-old male with a history of alcoholic cirrhosis and achalasia status post-Heller myotomy in 2019 presented to the emergency room (ER) following four episodes of vomiting bright red blood over the course of 12 hours. He also endorsed mild epigastric pain and loose dark stools. The patient was hypotensive with a blood pressure of 87/61, tachycardic to 112 beats per minute, afebrile, and with an oxygen saturation of 99% on room air. Digital rectal exam revealed melena. Hemoglobin was 8.3 g/dL, which was significantly decreased from the patient’s known baseline of approximately 14 g/dL. The patient continued to have episodes of hematemesis in the ER. Of note, the patient underwent an EGD only a month earlier for persistent dysphagia. EGD at that time showed a known diverticulum at the gastroesophageal (GE) junction, and there was no evidence of esophageal varices, as seen in Figure 1. The patient was treated with IV fluids, three units of blood, pantoprazole, and ceftriaxone and was started on an octreotide drip. He then underwent an EGD, which showed three columns of esophageal varices in the lower third of the esophagus. Although no red whale sign was present, there was a white nipple sign in the lower esophagus, indicating recent bleeding, as seen in Figure 2. Two bands were placed on the area with the white nipple sign. Following his EGD, the patient experienced no recurrent bleeding.

## Discussion

Esophageal varices are present in 50-60% of patients with compensated cirrhosis [3]. An initial bleeding episode associated with ruptured varices is associated with a mortality rate as high as 40% [4]. Therefore, screening guidelines are in place to ensure that patients with compensated cirrhosis undergo endoscopic surveillance for GE varices. According to the American Association for the Study of Liver Diseases (AASLD), an initial screening endoscopy should be performed at the time of diagnosis of cirrhosis [5]. If small varices are seen on the initial endoscopy, then endoscopy should be repeated every 1-2 years, but if no varices are seen on the initial endoscopy, then endoscopy should be repeated every 2-3 years [6]. Current guidelines do not recommend more frequent endoscopic surveillance, as the risk of variceal hemorrhage within three years of initial endoscopy in patients without varices is below 10% [7]. 

In our patient, an initial endoscopy showed no evidence of varices in the esophagus. As per current AASLD guidelines, repeat endoscopy for variceal screening wouldn’t be due for at least another two years. Yet, only 36 days after his initial endoscopy, the patient developed three columns of esophageal varices which had ruptured, causing hemorrhagic shock. Rapid development of esophageal varices has been reported throughout the literature, but typically in the setting of a potentially inciting factor [8]. For example, Majmudar et al. [9] reported a patient who had undergone placement of a left ventricular assist device (LVAD) and developed ruptured esophageal varices only 21 days after an initial endoscopy prior to the LVAD placement showed no varices. Furusawa et al. [10] reported a patient who presented with ruptured varices after only nine months of both a normal endoscopy and initiation of atezolizumab-bevacizumab for hepatocellular carcinoma. Finally, Shibata et al. [11] presented a patient who developed ruptured esophageal varices only 40 days after an initial endoscopy revealed no varices, during which time the patient developed acute cholangitis.

There were no identifiable potential inciting factors that took place within the 36 days between our patient’s initial endoscopy and his presentation with ruptured varices. However, the patient did have a history of achalasia and was surgically treated with a Heller myotomy back in 2019. The Heller myotomy involves cutting through the outer longitudinal muscle fibers of the lower esophagus. This extensive manipulation of the lower esophagus may have played a role in the rapid development of esophageal varices in the lower esophagus three years later. Within the literature, although there are several reports of managing achalasia with myotomies in patients with a history of esophageal varices [12], there are no known studies or reports of esophageal varices developing in patients with a history of myotomy.

## Conclusions

The rapid development of ruptured esophageal varices within only 36 days is quite uncommon. Current guidelines recommend repeat endoscopic surveillance in compensated cirrhotic patients to be every 2-3 years. Our patient’s history of a Heller myotomy may have played a role in the pathogenesis of his esophageal varices. As studies and reports regarding the association between myotomies and the development of esophageal varices are quite scarce, further literature on the topic is most certainly warranted.
